# Primary Antiphospholipid Syndrome Associated with Pneumonia: A Case Report of a 16-Year-Old Male Patient

**DOI:** 10.1155/2015/249612

**Published:** 2015-03-22

**Authors:** Süreyya Yilmaz, Fusun Topcu, Hadice Selimoglu Sen, Yasar Yildirim, Zülfükar Yilmaz, Ali Veysel Kara, Cihan Akgul Ozmen

**Affiliations:** ^1^Department of Chest Diseases, Faculty of Medicine, Dicle University, 21100 Diyarbakir, Turkey; ^2^Department of Internal Medicine, Faculty of Medicine, Dicle University, 21100 Diyarbakir, Turkey; ^3^Department of Radiodiagnostics, Faculty of Medicine, Dicle University, 21100 Diyarbakir, Turkey

## Abstract

Antiphospholipid syndrome (APS) is an autoimmune disease characterised by arterial and/or venous thrombosis and/or recurrent pregnancy loss in the presence of antiphospholipid (APL) antibodies. It is evaluated as APS when it develops associated with other systemic autoimmune diseases or primary APS if there is no concomitant disorder. In this study, we present a case of a 16-year-old male patient with primary APS. The patient was admitted with presumptive diagnosis of pneumonia, but multiple pulmonary thromboembolism (PTE) was observed on computerized tomography (CT) pulmonary angiography. APL antibodies positivity and thrombocytopenia developed in our patient. The patient was evaluated as primary APS since another etiology that could explain PTE was not found. Primary APS is a rare disease in children along with adolescents, compared with APS associated with other systemic autoimmune diseases. We present here a young male patient with primary APS and PTE to contribute to the literature. The patient initially had pneumonia but later developed PTE and thrombocytopenia.

## 1. Introduction

Antiphospholipid syndrome (APS) is an autoimmune disease characterised by arterial and/or venous thrombosis and/or recurrent pregnancy loss in the presence of antiphospholipid antibodies (anticardiolipin antibody (ACA), lupus anticoagulant (LAC)) [1]. Thrombosis in APS can be seen in both arterial and venous structures, and it has also been reported to occur in almost all parts of the vascular system. The most common type of APS-related venous thrombosis is deep vein thrombosis (DVT) in lower extremities with or without PTE [2]. It is evaluated as APS when it is associated with other systemic autoimmune diseases or primary APS if there is no concomitant disorder [1]. In order to make a diagnosis of APS associated with other systemic autoimmune diseases, there must be an autoimmune disease, in particular, systemic lupus erythematosus (SLE) [3]. Although the exact pathogenesis of the syndrome has not been elucidated, LAC and ACA are thought to be responsible [4]. Some infections have been reported to be likely to trigger the production of these antibodies, and infections can also be associated with thrombotic events in patients with APS [5].

## 2. Case Report

A 16-year-old male patient was admitted to our clinic with complaints of chest pain, fever, cough, and shortness of breath lasting for ten days, without any previous complaints. On physical examination, general condition was good and vital signs were stable. Head, neck, cardiovascular, gastrointestinal, extremities, and skin examinations were within normal limits. On respiratory system examination by listening, diminished breath sounds in bilateral lower lung zones and minimal crackles in some areas were determined.

In laboratory tests, hemoglobin, white blood cell (WBC), and thrombocyte counts were 14.6 g/dL, 19.4 K/UL, and 340 × 10^9^/L, respectively. C-reactive protein (CRP) and erythrocyte sedimentation rate (ESR) were, respectively, 40.7 mg/dL and 18 mm/hr. Anti-HIV was found negative. Arterial blood gas and biochemical analyses were all within normal limits.

**Figure 1:**
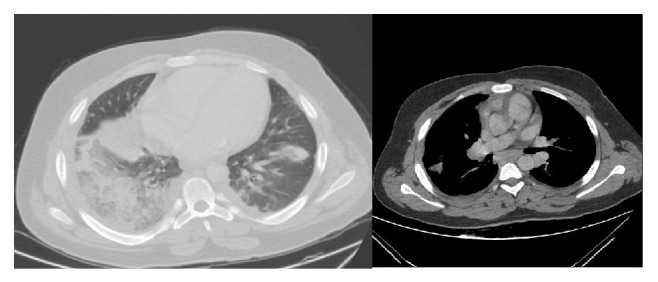
Consolidation areas in the lower lobe of right lung, upper-lower lobe of left lung, and lingula were observed in first drawn contrast enhanced computed tomography.

**Figure 2:**
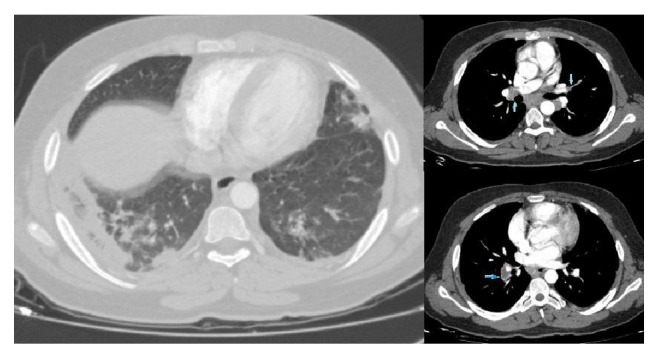
PTE was detected on the right main pulmonary artery and left pulmonary artery branches and consolidation areas in bilateral lower lobe and areas of ground glass in the upper and lower lobes in computed tomography pulmonary angiography.

The first contrast-enhanced thorax tomography of the patient revealed widespread consolidation and ground-glass appearance in the lower lobe of the right lung and consolidation areas that occurred by merging of nodules in the apicoposterior part of the left lung. Atelectasis and increased density due to consolidation were observed in the upper lobe lingula and lower lobe of the left lung. Patient was evaluated as pneumonia (Figure 1). Pulmonary embolism was not detected in this tomography. Antibiotics and general supportive therapy were started. Control thorax tomography was performed 25 days after the treatment since there was no improvement in the patient's cough, chest pain, and radiological status. Multiple hypodense filling defects were observed in the right main pulmonary artery and branches of left pulmonary artery at control computerized tomographic pulmonary angiography, and it was then evaluated as PTE. Patchy areas of consolidation in the lower lobes of both lungs were evaluated as necrosis and ground glass areas of the superior of the upper and lower lobes were evaluated as infiltration (Figure 2).

Treatment of the patient was revised. Anticoagulant therapy was started, and further examinations were performed. Collagen tissue disease panel results were found to be negative (ANA, P-ANCA, C-ANCA, anti-dsDNA, anti-Jо1, ant-Sm, anti-Sm/RNP, anti-Scl-70, anticentromere B, and anti-SSA). Complement components C3 and C4 were within normal limits. Thrombophilia panel was performed; anticardiolipin antibodies and lupus anticoagulant were found to be positive twice with an interval of 13 weeks, and activated partial thromboplastin time (aPTT) was prolonged. Thrombocytopenia developed on the second admission (108 × 10^9^/L) (Table 1). Deep venous thrombosis was not detected in Doppler ultrasonography performed on bilateral lower extremity venous. Transthoracic echocardiogram was normal.

## 3. Discussion

Antiphospholipid syndrome is a disease that emerged first with laboratory features. In the first studies conducted in the 1950s, an in vitro anticoagulant (lupus anticoagulant) that circulates in the blood of patients with SLE was observed, and this anticoagulant was found to be associated with a false positive VDRL test [6, 7]. Diagnosis of this syndrome, first described by Hughes in 1983, was made by the presence of thrombosis, recurrent miscarriages, and antiphospholipid antibodies (APL).

For the diagnosis of APS to be made, one clinical event, that is, thrombosis or recurrent miscarriages, and the presence of LAC or ACA IgG or IgM in the plasma on two or more occasions at least six weeks apart are required, according to the 1999 internal consensus statement diagnostic criteria [8]. In our case, LAC and ACA antibodies positivity with an interval of 13 weeks and demonstration of pulmonary thromboembolism met the diagnostic criteria of APS. The lack of the history of drug use and accompanying autoimmune disease also support the diagnosis of primary APS. Primary APS is a diagnosis of exclusion; hence, exclusion criteria can be used for diagnosis [9]. Clinical signs, such as rash (malar, discoid, and mucosal), arthritis, pleuritis, pericarditis, or laboratory signs, such as proteinuria, lymphopenia, anti-dsDNA, anti-ANA positivity, and drug usage history that can cause APL antibodies, were not found in our case. Therefore, he was diagnosed with primary APS.

APS is a relatively common cause of acquired venous thrombosis. More than 20% of DVT with or without pulmonary thromboembolism might be related to APL antibodies [2]. In addition, lupus anticoagulant is considered positive if Lac S/Lac C (Lac Screen/Lac Confirm) is found to be >1.2 [10]. Prolongation of aPTT has been found as another laboratory sign in these patients. In our case, LAC positivity was detected (Lac S/Lac C = 1.89), and also aPTT was prolonged.

In particular, ACA-IgG type is known to be responsible for thrombotic events [11]. In a previous study conducted with 56 cases, ACA-IgG level > 40 GPL-U was found to be the independent risk factor for thrombosis [12]. In our patient, ACA IgG level was detected as 72.6 U/mL. Medium or high degree of ACA IgG positivity is found in most APS patients. Both ACA IgG and ACA IgM levels were positive in our patient, but IgG levels were approximately three times as much as IgM levels.

The mean age of primary APS patients has been reported to be at around the age of 35–40 in previous studies [13, 14]. Primary APS is a rare condition in children, and the real prevalence is not known for childhood [15]. However, in a study carried out by Cervera et al., prevalence of APS occurring before the age of 15 was found as 2.8% [16]. Female/male ratio in primary APS is 5/1 [17]. Thrombocytopenia is observed in 20%–40% of patients with primary APS [18]. Thrombocytopenia is defined as platelet count <150 × 10^9^/L [12]. In our case, minimal degree thrombocytopenia was detected as (108 × 10^9^/L). Our patient is within childhood age limit, male, and he was initially diagnosed with pneumonia and thereafter with PTE and thrombocytopenia. Because of this rare condition, we were encouraged to do further research.

Many clinical signs can be seen in primary APS. In a previous cohort of 1000 patients with APS, DVT (31.7%), thrombocytopenia (21.9%), and pulmonary embolism (9.0%) have been observed [16]. Venous thrombosis, which is mostly seen in lower limbs, and secondly pulmonary embolism have been diagnosed in 47.2% of patients with primary APS [13]. The most common sign observed in APS is recurrent DVT; 50% of DVT cases may be accompanied by pulmonary embolism. Pulmonary embolism may be the first clinical sign, as in our patient [3]. Episodes of thrombosis can be spontaneous or develop as a result of conditions such as traumas, operations, immobilization, venous stasis, and usage of oral contraceptives [19]. Pneumonia is recognized to be associated with APS [5], and it is well known that infections are common triggers of catastrophic and transient APS [20]. Pneumonia may have triggered pulmonary thromboembolism in our patient; however, he was not diagnosed as catastrophic APS.

Primary APS is a rare disease in children as well as adolescents, compared with APS associated with other systemic autoimmune diseases. Primary APS triggered by pneumonia and resulting in pulmonary thromboembolism in a 16-year-old male patient is a rarely seen condition, so we present this case report in order to contribute to the literature.

## Figures and Tables

**Table 1:** Thrombophilia panel of patient.

Parameters	Initially	13th week	References
Lac Confirm	66.2 sec	58.1 sec	30–38
Lac Screen	125 sec	102 sec	31–44
Lac S/Lac C	1.89	1.76	0.8–1.2
AT 3 activity	89%	—	84.6–120
F VIII	36.8%	—	50–150
Anticardiolipin IgM	22.6 U/mL	17.2 U/mL	0–7
Anticardiolipin IgG	72.6 U/mL	60.3 U/mL	0–10
aPTT	38 sec	48.6 sec	25–35
INR	1.1	2.1	0.88–1.2
PAI-1 (4G/5G)	Heterozygous	—	
MTHFR (C677T)	Heterozygous	—	
MTHFR (A1298C)	Normal	—	
F XIII	Normal	—	
F II (G20210A)	Heterozygous	—	
F V Leiden	Normal	—	
Protein C	Normal	—	
Protein S	Normal	—	
Platelet count	108 × 10^9^/L	255 × 10^9^/L	142–424

Lac: Lupus anticoagulant, MTHFR: methylenetetrahydrofolate reductase, F: factor, and PAI: plasminogen activator inhibitor.
